# Three Different Interaction Patterns between MCM-41 and Proteins

**DOI:** 10.3390/ijms232415850

**Published:** 2022-12-13

**Authors:** Yuke Xie, Ziqiao Zhong, Wenhao Wang, Ying Huang, Chuanbin Wu, Xin Pan, Zhengwei Huang

**Affiliations:** 1 College of Pharmacy, Jinan University, Guangzhou 511443, China; 2 School of Pharmaceutical Sciences, Sun Yat-Sen University, Guangzhou 510006, China

**Keywords:** MCM-41, nanoparticle adsorption profile, protein structure, interaction patterns

## Abstract

As one of the most studied mesoporous silica nanoparticles (MSNs) in drug delivery systems, Mobil Composition of Matter No. 41 (MCM-41) possesses unique properties including perfect channel architecture, excellent load capacity, and good biocompatibility. However, the applications of MCM-41 nanoparticles in drug delivery have not yet been industrialized, due to the interaction between MCM-41 and biomolecules (especially proteins) that affect their in vivo behaviors after dosing. To investigate the interactions between MCM-41 and proteins, this study selected bovine serum albumin (BSA), lysozyme (Lyso), and bovine hemoglobin (BHb) as model proteins and characterized the ultraviolet-visible, fluorescence, circular dichroism spectra and the protein adsorption of MCM-41-protein complex. The UV-Vis spectra exhibited the different absorption increment degrees of three proteins. The fluorescence spectra showed that the fluorescence intensity of proteins changed by different trends. The CD spectra indicated that the secondary structure changes were ranked as BSA > Lyso > BHb, which is consistent with the protein’s adsorption capability on MCM-41. It was shown that there were three different patterns of MCM-41-proteins interactions. The hydrophilic and low-charged BSA followed the strong interaction pattern, the hydrophilic but heavily charged Lyso followed the moderate interaction pattern, and the hydrophobic BHb followed the weak interaction pattern. Different interaction patterns would lead to different effects on the structural properties of proteins, the surface chemistry of MCM-41, and the absorption capability of proteins on MCM-41. We believe our study will provide a better insight into the application of MCM-41 nanoparticles in drug delivery systems.

## 1. Introduction

With the rapid development of nanomaterials, increasing interest in mesoporous silica nanoparticles (MSNs) as drug delivery systems have been observed in the past several decades [1]. Due to their unique characteristics for drug delivery, such as large surface area, controllable particle size and shape, huge load capacity and biocompatibility, extensive research has been carried out on the biomedical applications of MSNs [2]. Especially, due to their high density of surface silanol groups, MSNs can be chemically modified with various components and potentially used in sustained, controlled, and targeted delivery [3]. For instance, they are exploited for antitumor therapy [4] and the treatment of different bone diseases [5]. Therefore, MSNs are the ideal and promising therapeutic nanocarriers for drug delivery.

Mobil Composition of Matter No. 41 (MCM-41) is regarded as one of the most studied MSNs in drug delivery systems. Equipped with a two-dimensional hexagonal structure, the MCM-41 possesses many advantages as a nanocarrier, including high thermal stability, perfect channel architecture, and narrow pore size distribution [3], which is considered as a very well-organized homogeneous functional material [6]. Additionally, drug molecules with multiple sizes, shapes, and functions have been proved to be well-suited for encapsulating in MCM-41 [7,8]. Some examples of applying MCM-41 in biomedicine are as follows. In 2001, Vallet-Regi and co-workers found that MCM-41 has the ability to recognize and deliver organic compounds including the nonsteroidal anti-inflammatory drug ibuprofen [9]. Zeng and co-workers successfully used MCM-41 as the controlled drug delivery system of aspirin [10]. Zhang and co-workers encapsulated the natural active ingredient Hypocrellin A in the MCM-41 and performed the spectroscopic studies [11]. Additionally, MCM-41 was also applied in the inclusion of medicative proteins, such as cytochrome c and myoglobin [12,13]. Therefore, MCM-41 has shown great application prospects, pending translation.

However, the applications of MCM-41 in drug delivery systems have not yet been industrialized presently. It may be mainly due to the unknown in vivo fate. When nanoparticles like MCM-41 were administrated in vivo, different biomolecules, such as proteins, lipids, nucleic acids, and carbohydrates, may interact with nanoparticles and immediately be adsorbed on their surface [14]. Particularly, most nanoparticles are rapidly coated by proteins and formed protein corona when they contact biological environments [15]. The nanoparticle-protein interaction has been shown to lead to reduced cellular uptake [16], affecting the volume of distribution, organ disposition, and metabolism of administrated nanoparticles [15], and causing loss of specificity in targeting [17], which may result in some negative influences on the therapeutic effect of nanoparticles. Moreover, most proteins adsorbed on the nanoparticle surface will enhance immunogenicity [14] or even cause inflammation [18], provoking safety issues. Overall, through the nanoparticle-protein interaction, the biophysical properties of the nanoparticles can be stunningly changed [19], and nanoparticles may be conferred a new biological identity in the biological environment [18], which will make the in vivo behaviors of nanoparticles more unpredictable and uncontrollable. Hence, the nanoparticle-protein interaction is considered as the key element that influences how nanoparticles behave in the biological system. Thus, it is critical to investigate and clarify the interactions between MCM-41 and proteins.

In this work, bovine serum albumin (BSA), lysozyme (Lyso) and bovine hemoglobin (BHb) were selected as model proteins and incubated with different concentrations of MCM-41 nanoparticles. These proteins were widely used in the interaction research as acidic protein, basic protein and neutral protein, respectively. Through measuring ultraviolet-visible (UV-Vis) absorption spectra, fluorescence spectra, circular dichroism (CD) spectra and protein adsorption capacity, different patterns of MCM-41-protein interaction were expounded and discussed. This study will provide new insights into the interaction between MCM-41 and proteins, as well as a reference on MCM-41 applications in nano-drug delivery systems.

## 2. Results and Discussion

### 2.1. Preparation of MCM-41 Nanoparticles

**Figure 1:**
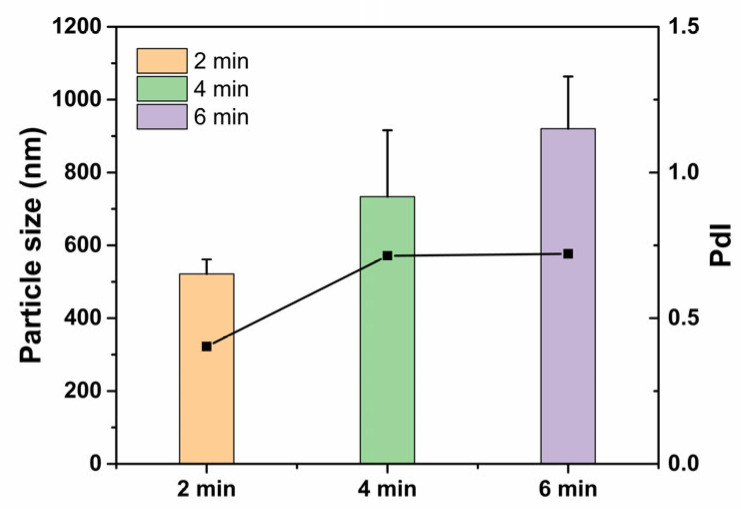
Particle size (column) and PDI (line) of MCM-41 nanoparticles with different ultrasonication times.

MCM-41 dispersion was prepared by an ultrasonication method, and the particle size and polydispersity index (PDI) were determined to select the proper ultrasonication time. As shown in Figure 1, with ultrasonication for 2 min, the particle size of MCM-41 was 521.6 ± 39.4 nm, with a lower PDI value (Figure S1). When the time was higher than 2 min, it showed excessive particle size and the PDI value was much higher. Therefore, the ultrasonication time was chosen as 2 min for the follow-up study.

### 2.2. UV-Vis Absorption Spectra of the Interaction between MCM-41 Nanoparticles and Proteins

The UV-Vis absorption spectra of MCM-41 incubated with proteins were measured to provide the adsorption information of proteins on nanoparticles [18]. Due to the existence of acid amino residues with a characterized UV-Vis absorbance peak like tryptophan (Trp), the absorbance value (Abs) and the peak wavelength of different proteins were analyzed to analyze the nanoparticle–protein interaction. The Abs of MCM-41 was small and had little effect on the Abs of MCM-41 after incubation with protein (Figure S2).

**Figure 2:**
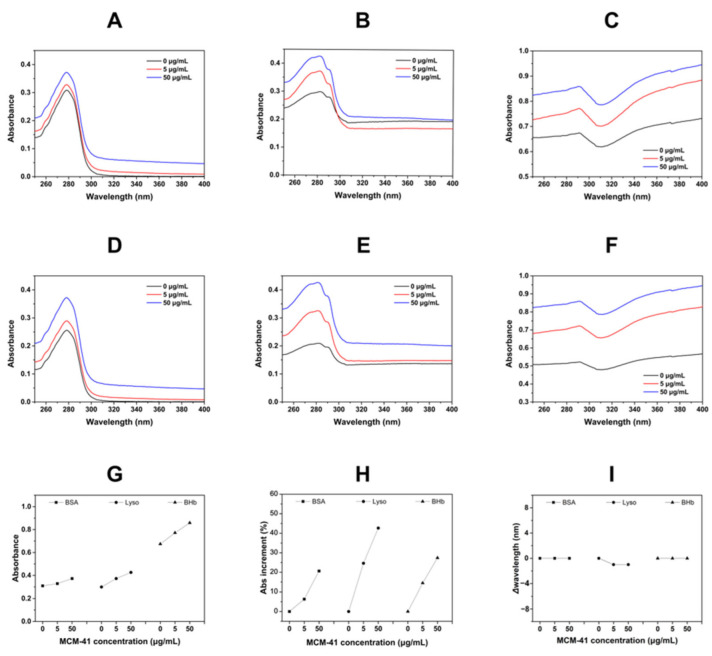
(**A**–**C**) UV-Vis spectra of MCM-41 incubated with BSA (**A**), Lyso (**B**) and BHb (**C**). (**D**–**F**) The normalized UV spectra: BSA (**D**), Lyso (**E**) and BHb (**F**). (**G**) The Abs increment of MCM-41 was incubated with different proteins. (**H**) The percentage of Abs intensity increment of different proteins incubated with MCM-41. (**I**) The *Δ*wavelength of different proteins incubated with MCM-41.

As shown in Figure 2, when BSA, Lyso, and BHb were incubated with MCM-41, the Abs of the three proteins was all increasing with the increment of MCM-41 concentration. The Abs of BSA increased from 0.31 to 0.37, that of Lyso increased from 0.30 to 0.43 and that of BHb increased from 0.67 to 0.86. Figure 2D–F showed the normalized UV spectra. There was no significant change in the peak wavelength of three proteins incubated with MCM-41 (Figure 2I). However, the percentages of Abs increment of BSA, Lyso, and BHb were different (Figure 2H). The Abs increments of proteins were related to the exposure of protein chromophores. The proteins’ interaction with MCM-41 might have led to the exposure of chromophores that were originally hidden inside the proteins, and with the increase of MCM-41 concentration, this interaction was intensified, leading to more exposure of chromophores, and thus, UV absorption was increased. The above results indicated that there might be different interactions between MCM-41 and three proteins.

### 2.3. Fluorescence Spectra of the Interaction between MCM-41 Nanoparticle and Proteins

**Figure 3:**
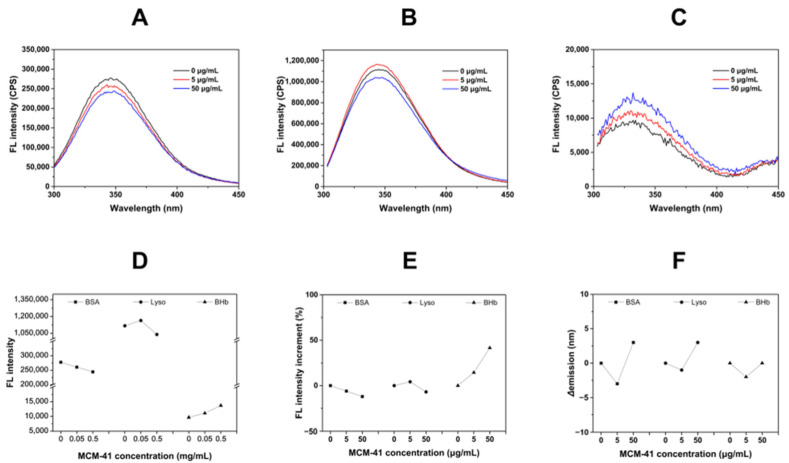
(**A**–**C**) Fluorescence spectra of MCM-41 incubated with BSA (**A**), Lyso (**B**) and BHb (**C**). (**D**) The fluorescence intensity increments of different proteins which were incubated with MCM-41. (**E**) The percentage of fluorescence intensity increment of different proteins incubated with MCM-41. (**F**) The Δemission of different proteins incubated with MCM-41.

To explore the intrinsic fluorescence changes of proteins due to the interaction between the MCM-41 and proteins, the fluorescence spectra were recorded, which was widely used to analyze the intensity of the nanoparticle-protein interaction. As the three model proteins used in this study all contained natural fluorescent amino acid residues (like Trp), when the protein interacted with MCM-41 and the protein conformation altered, the corresponding fluorescence intensity and the emission wavelength would change [20]. As illustrated in Figure 3, after interacting with different concentrations of MCM-41, the fluorescence intensity of BSA, Lyso and BHb had different alterations. The fluorescence intensity of BSA significantly decreased from 277,500 to 244,120 with the increment of MCM-41 concentration (Figure 3A). It was indicated that MCM-41 could lead to the fluorescence quenching of BSA, and the degree of fluorescence quenching was positively correlated with the concentration of MCM-41. Interestingly, when the MCM-41 concentration was at 5 μg/mL, the fluorescence of Lyso showed a slight increase, while the fluorescence intensity of Lyso decreased from 1,116,860 to 1,039,620 when the concentration of MCM-41 was 50 μg/mL (Figure 3B). This suggested that MCM-41 at higher concentrations could have a strong interaction with Lyso, which resulted in fluorescence quenching, whereas at low concentrations, there were no such significant effects. The formation of MCM-41-proteins complexes caused fluorescence quenching, and the degree of quenching was proportional to the number of bound proteins [21]. Thus, the maximum degree of quenching of different proteins was compared. As shown in Figure 3E, when the MCM-41 concentration was at 5 μg/mL, the fluorescence quenching of BSA occurred while Lyso did not, and with MCM-41 concentration set as 50 μg/mL, the fluorescence intensity decrement of BSA was about 12%, higher than that of Lyso (6.9%), which indicates that the interaction between BSA and MCM-41 was stronger than that of Lyso. However, with the increasing concentration of MCM-41, the fluorescence intensity of BHb increased from 9680 to 13,700 (Figure 3C), indicating the interaction pattern with MCM-41 of BHb was different from that of BSA and Lyso.

Then, the changes in emission wavelength (*Δ*emission) were summarized in Figure 2F. A noticeable red-shift (3 nm) was observed in the fluorescence emission spectra of BSA and Lyso with 50 μg/mL MCM-41. The change in the surrounding microenvironment of Trp residues might be responsible for this type of peak shift [21]. It was suggested that BSA and Lyso had undergone conformational changes, affecting the microenvironment in which the Trp residues of the protein were located. The red-shift indicated that the tryptophan residues in BSA and Lyso were in a more hydrophilic environment after interaction with MCM-41, which could be due to BSA and Lyso adsorption on the hydrophilic surface of MCM-41. Since BSA and Lyso adsorbed on the hydrophilic surface of MCM-41, the polarity of the hydrophobic tryptophan microenvironment changed as the protein conformation changed. In contrast, the fluorescence emission of BHb did not appear red-shift and Figure 3C showed that the fluorescence intensity of BHb increased with the concentration of MCM-41. It was indicated that the MCM-41-BHb interaction could not lead to changes in the Trp residue microenvironment and fluorescence quenching, demonstrating that BHb might not be tightly adsorbed on the hydrophilic surface of MCM-41 and have the weaker interaction with MCM-41 than BSA and Lyso. Hence, it was suggested that the interaction force followed the rank of MCM-41-BSA > MCM-41-Lyso > MCM-41-BHb. In summary, the interactions between MCM-41 and the three proteins were different, which led to different behaviors of fluorescence.

### 2.4. CD Spectra of the Interaction between MCM-41 and Proteins

**Figure 4:**
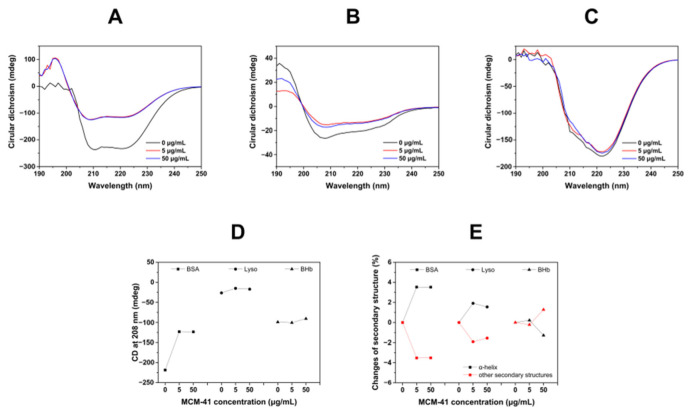
(**A**–**C**) CD spectra of MCM-41 incubated with BSA (**A**), Lyso (**B**) and BHb (**C**). (**D**) The CD ellipticity values at 208 nm of different proteins incubated with MCM-41. (**E**) The changes of α-helix and other secondary structures’ contents of different proteins incubated with MCM-41.

Moreover, changes in the conformational structure of proteins adsorbed on the surface of MCM-41 were investigated by CD spectra. CD spectroscopy had been widely used to analyze changes in protein secondary structure [22]. For example, Andra et al. investigated the secondary structure rearrangement of BSA after interaction with GO-PAMAM using CD spectroscopy. Additionally, they found that this interaction promoted the rearrangement of the protein backbone, leading to strongly twisted β-sheet secondary structure architecture [23]. As shown in Figure 4, we could observe two negative bands at 208 and 220 nm, the values of which were considered characteristic absorption peaks of the α-helix of protein. Then, the ellipticity value at 208 nm was summarized in Figure 3D. As shown in Figure 4A, after incubating with MCM-41, the absorption intensity of the CD band changed significantly and the ellipticity value at 208 nm was increased from −218.6 to −123.5, indicating that the interaction between MCM-41 and BSA could induce significant changes in the secondary structure of BSA. However, when Lyso and BHb were incubated with MCM-41, moderate intensity changes of Lyso (Figure 4B) and slight changes of BHb (Figure 4C) could be observed, which suggested that MCM-41 could change the secondary structure of Lyso to a moderate extent and that of BHb to a low extent. The changes of secondary structures calculated by the formula were also consistent with the above results (Figure 4E). Therefore, it was indicated that three proteins had different interaction levels with MCM-41, and it was reasonable that the intensity of interaction between proteins and MCM-41 ranked as BSA > Lyso > BHb.

### 2.5. Protein Adsorption Assay

**Figure 5:**
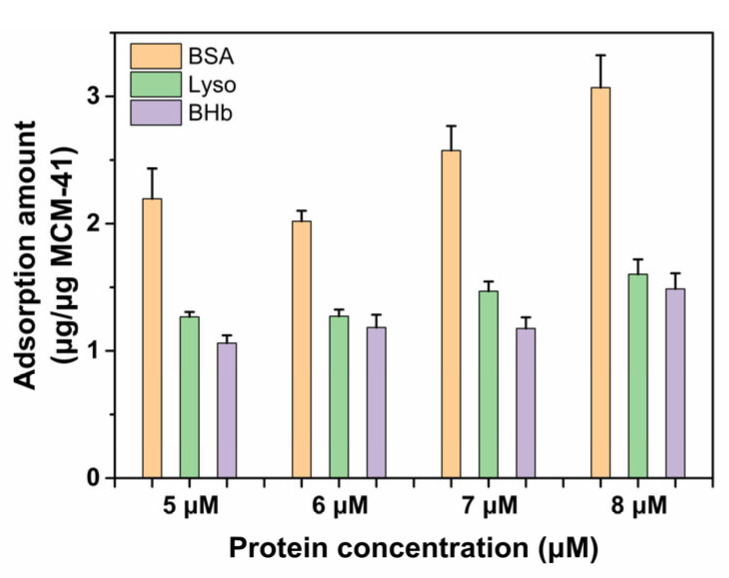
The adsorption amount of different proteins on MCM-41 at different concentrations.

According to the above results, due to the different intensities of interaction between proteins and MCM-41, the absorption capability of proteins on MCM-41 was expected to be BSA > Lyso > BHb. To testify different adsorption capabilities of MCM-41 for three proteins, a BCA assay was used to measure the protein adsorption amount. As shown in Figure 5, the adsorption amount of BSA was much higher than Lyso and BHb, as expected. As for BSA, the largest adsorption amount was about 3.07 μg/μg MCM-41, 1.92 times higher than Lyso and 2.07 times higher than BHb, while the value was measured as 1.60 μg/μg MCM-41 for Lyso, 1.08 times higher than BHb. Hence, the adsorption capability on MCM-41 was BSA > Lyso > BHb, which was consistent with the intensity of interaction shown by fluorescence and CD spectra. In conclusion, these findings jointly indicated that there might be different interaction patterns between proteins and MCM-41.

### 2.6. Interaction Patterns

As shown by the results above, the interaction between MCM-41 and the three proteins was different. Additionally, the results of various tests were summarized in Table 1.

**Figure 6:**
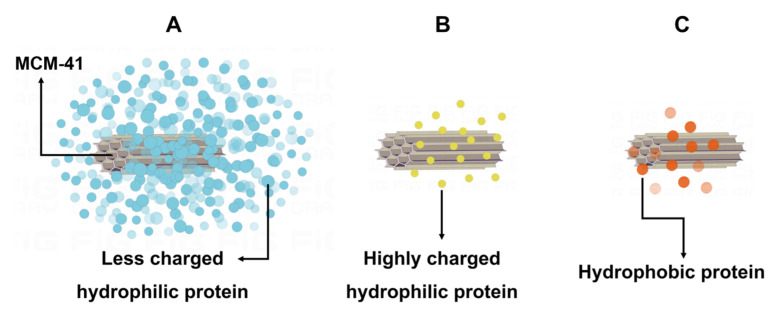
(**A**) The strong interaction pattern. (**B**) The moderate interaction pattern. (**C**) The weak interaction pattern.

According to the above results, we hypothesized that there might be three patterns of interaction between MCM-41 and proteins (Figure 6). Firstly, BSA might follow a strong interaction pattern. It might have a strong interaction with MCM-41, which would decrease the fluorescence intensity of BSA and form a quenching complex, and as the concentration of MCM-41 increased, the amounts of bound proteins would increase, and the degree of fluorescence quenching would also increase. Additionally, this strong interaction pattern might lead to the results of strong secondary structure changes of BSA and its high adsorption capability on MCM-41. Secondly, Lyso might follow the moderate interaction pattern. It might interact with MCM-41 with moderate intensity, and only in the presence of high concentrations of MCM-41 could the fluorescence intensity of Lyso be inhibited, resulting in fluorescence quenching of Lyso. Additionally, this moderate interaction pattern might cause the consequences that the secondary structure change of Lyso was medium and its adsorption capability on MCM-41 was lower than BSA. Thirdly, BHb might follow a weak interaction pattern. It might have a weak interaction with MCM-41, the fluorescence intensity of BHb would not be suppressed without the formation of a quenching complex, and the fluorescence intensity would increase with the increase of MCM-41 concentration. Additionally, this weak pattern might result in smaller changes in BHb secondary structure and its weaker adsorption capability.

The hydrophobic interaction and electrostatic interaction could be used as the main elements to explain these three interaction patterns. For better interpretation, the molecular weight (MW), isoelectric point (PI), grand average of hydropathicity (GRAVY), and the amino acid residue number of BSA, Lyso and BHb were summarized in Table 2.

For one thing, the hydrophilicity of proteins could influence the interaction between the proteins and MCM-41 [24]. Through the GRAVY of BSA, Lyso and BHb, the holistic hydrophilicity of proteins could be quantitatively analyzed. Because the hydrophobic proteins had positive GRAVY value while hydrophilic proteins had negative value, BSA and Lyso were hydrophilic and BHb was hydrophobic. The surface of MCM-41 was highly hydrophilic due to the large number of silanol groups on its surface, which resulted in the hydrophilic proteins BSA and Lyso could on the surface of MCM-41 being easily absorbed, whereas it was hard for hydrophobic protein BHb to adsorb on the surface of MCM-41. This reasonably explained the phenomenon of fluorescence quenching and red-shift of emission wavelength after the BSA and Lyso interacted with MCM-41, as well as the weaker interaction between BHb and MCM-41.

For another thing, through the analysis of the charge performance of the protein, the differences in the interactions of BSA and Lyso with MCM-41 could be better explained. The pH of ultrapure water is 6.9 and the isoelectric point (PI) of MCM-41 is 3.42~3.77 [25]. When MCM-41 was suspended in ultrapure water, its surface was negatively charged. On the one hand, because the pH of the ultrapure water was close to the PI of BSA, BSA carried less charge, and it could be more tightly adsorbed and compacted on the surface of MCM-41. Adsorption of BSA on the surface of MCM-41, in this case, might be primarily due to site-specific interactions caused by the presence of positive charge plaques on the surface of BSA, and this adsorption might be driven by structural changes in the adsorbed BSA [26]. It was consistent with the analysis of fluorescence results above. On the other hand, because the PI of Lyso is 10.8, it carried a significant positive charge in the solution. When a small amount of MCM-41 was added, due to the high concentration of protein in the solution at that time, a large number of positively charged Lyso in the solution could not be tightly adsorbed on the surface of MCM-41 because of the repulsion between Lyso–Lyso molecules. Additionally, compared with the interaction between the surface of the negatively charged MCM-41 and the surface of the positively charged protein, the lateral repulsion force between the protein molecules was more obvious. Vinu and coworkers came to a similar conclusion in their experiments [27]. As a result, when less MCM-41 was present, Lyso could not be well adsorbed on the surface of MCM-41. When MCM-41 concentrations were much higher, the interaction between Lyso and MCM-41 would be much stronger.

### 2.7. Inspiration for MCM-41 Drug Delivery Application

Herein, MCM-41 nanoparticles would interact with proteins. Among them, hydrophilic proteins (like BSA and Lyso) might have stronger interactions with MCM-41, adsorb better on the hydrophilic surface of MCM-41, and have a greater impact on the protein structure. However, for hydrophobic proteins (like BHb), the interaction with MCM-41 was weaker, it was more difficult to adsorb on the hydrophilic surface of MCM-41 and the effect on protein structure is smaller. When there was more charge on the surface of the protein, the repulsive effect between the proteins would weaken the adsorption and interaction of the protein to MCM-41.

Therefore, when MCM-41 nanoparticles were administrated in vivo, some of the issues that could arise from the interaction with proteins in biological fluids demanded our attention. According to the experimental results, MCM-41 might adsorb a large amount of serum albumin in the blood after injection administration, and the albumin was originally intended to maintain plasma colloidal osmolality [28]. Besides, as the natural bacteriostatic agent for the human body, Lyso could dissolve bacteria by damaging bacterial cell walls [29]. The interaction between MCM-41 and these proteins might affect their normal function. Furthermore, the strong interaction of proteins (especially the hydrophilic ones) with MCM-41 and their adsorption on its surface might cause the protein to denature and be excluded from the complement system [30], which affected the fate of MCM-41 in vivo.

To reduce the effect of MCM-41-protein interactions on MCM-41 in vivo fate, we could modify the surface of MCM-41 to reduce its adsorption of proteins by shielding modifications. Polyethylene glycol (PEG) and zwitterion modifications were mainly used as shielding modifications, and the PEGylation was usually used to reduce non-specific protein adsorption [31] and zwitterions could almost eliminate specific protein adsorption [32]. To sum up, this study was expected to deepen the understanding of the interaction between MCM-41 and proteins and provide a reference value for the surface modification of MCM-41 nanoparticles and their applications in nano-drug delivery systems.

## 3. Materials and Methods

### 3.1. Materials

MCM-41 was purchased from Jiangsu XFNANO Materials Tech. Co., Ltd. (Jiangsu, China). BSA was supplied by neoFroxx GmbH (Einhausen, Germany). Lysozyme was provided by YINGMAO Analysis and Testing Co., Ltd. (Guangzhou, China). BHb was purchased from Yuanye Bio-Technology Co., Ltd. (Shanghai, China). The bicinchoninic acid (BCA) protein assay kit was provided by Cwbiotech Co., Ltd. (Beijing, China). Ultrapure water was obtained by the VEOLIA-ELGA system (Veolia group, Paris, France).

### 3.2. Preparation of MCM-41 Nanoparticles

To prepare MCM-41 dispersion, MCM-41 (1 mg) was dissolved in 10 mL ultrapure water and sonicated by an Ultrasonic Cell Disruptor (Bilon Instrument Co. Ltd., Shanghai, China) in the ice water bath for 2, 4 or 6 min. Then, the MCM-41 dispersion was diluted with ultrapure water to obtain the MCM-41 nanoparticles at a concentration of 10 and 100 μg/mL.

### 3.3. Preparation of MCM-41 Nanoparticles Incubated with Proteins

The MCM-41 nanoparticles with different concentrations were mixed with different protein solutions (16 μM BSA, Lyso and BHb) in equal volumes and incubated at 37 °C and 150 rpm for 2 h. All proteins were dissolved in ultrapure water.

### 3.4. UV-Vis Absorption Spectra

To investigate the changes in UV absorption group exposure due to the interaction, the ultraviolet spectrophotometer (UV-2600, Shimadzu Co., Ltd., Kyoto, Japan) with 1.0 cm quartz cells was used to record the UV-Vis spectra. The UV spectra were recorded from 250 nm to 400 nm at room temperature with sampling points every 1 nm, with the ultrapure water used as the blank. The measurements were repeated three times in parallel.

### 3.5. Fluorescence Spectra

To explore the intrinsic fluorescence changes of proteins after the interaction, the fluorescence spectra of the different proteins that interacted with MCM-41 were measured by Fluoromax-4 (HORIBA, Ltd., Kyoto, Japan) using a 3 cm quartz cuvette. The different proteins (8 μM) were incubated with MCM-41 nanoparticles at concentrations of 5 and 50 μg/mL at 37 °C for 2 h. The emission spectra were recorded from 300 nm to 450 nm with a slit width of 3 nm, and excitation was performed at 280 nm with a slit width of 3 nm.

### 3.6. CD Spectra

To analyze changes in protein secondary structure, the CD spectra of MCM-41 nanoparticles incubated with proteins were recorded with a Chirascan spectropolarimeter (Applied Photophysics Ltd., Leatherhead, Surrey, UK) using a 1 mm path-length quartz cell at room temperature. CD spectra were measured from 190 to 250 nm with 1 nm bandwidth and under a nitrogen (N_2_) atmosphere. The measurements were repeated three times in parallel.

The percentage of α-helix content and other secondary structure contents could be calculated using the following equation [33,34]:

$$\alpha -Helix\left(\%\right)=\left[\frac{-{\mathrm{MRE}}_{208}-4000}{33000-4000}\right]\times 100$$

$${\mathrm{MRE}}_{208}=\frac{\mathrm{observed}\;\mathrm{CD}\left(\mathrm{mdeg}\right)}{C_{p}nl\times 10}$$

$$\mathrm{Others}\left(\%\right)=100\%-\alpha -Helix\left(\%\right)$$

*C_p_* is the molar concentration of the protein, *n* is the number of amino acid residues, and *l* is the path length (1.0 mm). Then, the changes in α-helix content and other secondary structure contents compared to pure protein were then calculated.

### 3.7. Protein Adsorption Assay

The protein adsorption was determined by BCA assay. Briefly, the MCM-41 nanoparticles were incubated with BSA, Lyso and BHb at different concentrations for 2 h. centrifuged using the ultrafiltration tube. The proteins not adsorbed were separated by ultrafiltration centrifugation at 4000 rpm for 30 min and collected for the BCA assay. Then, the protein adsorption amount was calculated. The measurements were repeated three times in parallel.

## 4. Conclusions

In this study, we investigated the interaction between MCM-41 nanoparticles and different proteins including BSA, Lyso and BHb. By analyzing the spectra performance and protein physicochemical properties, we supposed that the hydrophilicity and charge of proteins significantly impacted their adsorption behavior on MCM-41. The hydrophilic BSA and Lyso could have a stronger interaction with MCM-41, inducing the quenching of the intrinsic fluorescence of proteins. Among them, the stronger interaction was revealed by the less-charged BSA, which lead to the more obvious secondary structure change and larger adsorption amount. Whereas due to the less affinity with the highly hydrophilic silanol groups on MCM-41 surface, the hydrophobic BHb was proven to exert the weakest interaction with MCM-41 with no obvious change of fluorescence intensity and secondary structure change. In conclusion, the hydrophilicity and charge of proteins were important factors affecting the MCM-41-protein interaction pattern.

We believe that our research on MCM-41-protein interaction could assist in the development and application of MCM-41 in drug delivery systems. In the future, we will explore the interactions between MCM-41 and other proteins, investigate more types of silicon materials and promote meticulous cell and animal experiments.

## Figures and Tables

**Table 1:** The results of the study on MCM-41-proteins.

Test	Result	Conclusion
UV-Vis	The Abs increase of BHb was greater than BSA and lower than Lyso.	The interactions between MCM-41 and the three proteins were different.
Fluorescence	The fluorescence quenching degree of BSA was stronger than that of Lyso, and the fluorescence intensity of BHb increased.	The interaction force followed the rank of MCM-41-BSA > MCM-41-Lyso > MCM-41-BHb.
CD	The extent of secondary structure change was BSA > Lyso > BHb.	The intensity of interaction between proteins and MCM-41 was BSA > Lyso > BHb.
Protein adsorption	The adsorption amount of MCM-41 was BSA > Lyso > BHb.	The adsorption capability was BSA > Lyso > BHb.

**Table 2:** The physicochemical properties of proteins.

Protein	Molecular Weight (MW)	Isoelectric Point (PI)	Grand Average of Hydropathicity (GRAVY)	Amino Acid Residue Number
BSA	69,222	5.82	−0.433	583
Lyso	14,780	10.80	−0.153	129
BHb	64,690	6.80	0.004	141

The data are from ExPASy and Research Collaboratory for Structural Bioinformatics PDB.

## Data Availability

The data presented in this study are available on request from the corresponding author.

## Supplementary Materials

The following supporting information can be downloaded at: https://www.mdpi.com/article/10.3390/ijms232415850/s1, Figure S1: PDI of MCM-41 nanoparticles with different disruption time; Figure S2: UV-Vis spectra of 50 μg/mL MCM-41; the method for preliminary computation of protein secondary structures.
