# Supplementary material for: Three Different Interaction Patterns between MCM-41 and Proteins
Source: Int J Mol Sci. 2022 Dec 13;23(24):15850. doi: 10.3390/ijms232415850 (PMC9781137; doi:10.3390/ijms232415850)
Supplement: Supplementary file 1 [file ijms-23-15850-s001.zip › Supplementary Materials/Supporting information.pdf]

Three Different Interaction Patterns between MCM-41 and Proteins

Yuke Xie [1,†], Ziqiao Zhong [1,†], Wenhao Wang [2], Ying Huang [1,*], Chuanbin Wu [1], Xin Pan [2] and Zhengwei Huang [1,*]

1 College of Pharmacy, Jinan University, Guangzhou 511443, China; k1976624451@163.com (Y.X.); zhongzq@stu2022.jnu.edu.cn (Z.Z.); chuanbin_wu@126.com (C.W.)
2 School of Pharmaceutical Sciences, Sun Yat-Sen University, Guangzhou 510006, China; wangwh37@mail2.sysu.edu.cn (W.W.); panxin2@mail.sysu.edu.cn (X.P.)
* Correspondence: huangy2007@jnu.edu.cn (Y.H.); huangzhengw@jnu.edu.cn (Z.H.)
† These authors contributed equally to this work.

Supporting information

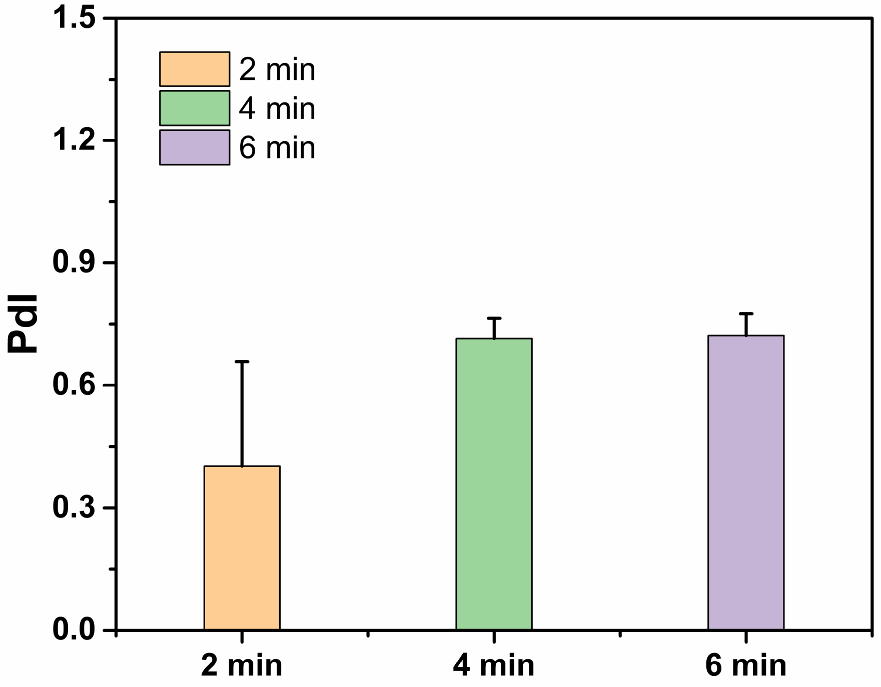

Figure S1. PDI of MCM-41 nanoparticles with different disruption time

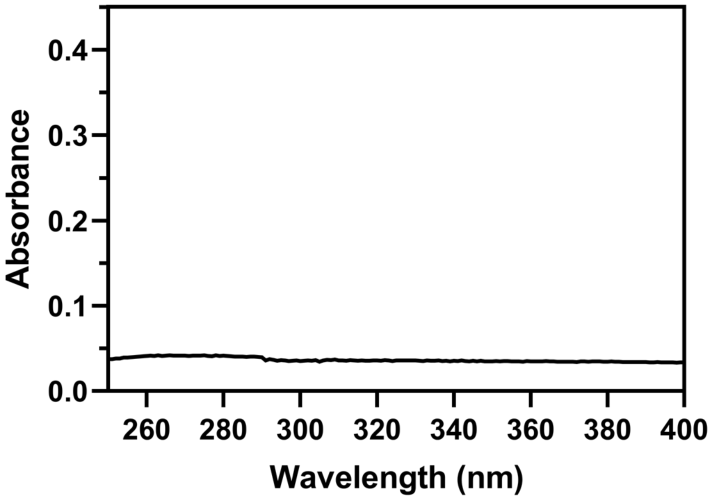

Figure S2. UV-Vis spectra of 50 μg/mL MCM-41

The following method was used to perform preliminary computational study:

First of all, we used the website https://www.uniprot.org/ and https://cactus.nci.nih.gov/translate/ to find protein amino acid sequence (FASTA format) and the structure of MCM-41 monomer (silicon dioxide, SMILES format), and transformed the above information into text files (.txt). Then we chose BioCPI function (CPI: chemical-protein interaction) in website http://biotriangle.scbdd.com/home/index/, which is a tool used for the calculation of chemical-protein interaction features. According to the prompts, we uploaded the .txt files to the corresponding column and started the simulation calculation. The default parameters were used, and the interaction features in model 1 and model 2 were obtained as comma-separated falues files (.csv). The detailed definition of model 1 and model 2 could be found in http://biotriangle.scbdd.com/static/media/BioTriangle_BioCPI.pdf. Microsoft Excel 2019 software was utilized as the reading medium for these .csv files, and we put them in the zip pack of Supplementary Materials.

According to the preliminary calculation results of interaction features, theoretical paremeters about protien-MCM-41 interactions were non-zero values. Thus, MCM-41 did interact with BSA, Lyso, and BHb. And the results of the preliminary computation would help us to carry out further visualized molecular docking.
